# Supplementary material for: Polygenic prediction of treatment efficacy with causal transfer learning
Source: medRxiv. 2025 Nov 1:2025.10.15.25338051. Preprint. [Version 2] doi: 10.1101/2025.10.15.25338051 (PMC12633083; doi:10.1101/2025.10.15.25338051)
Supplement: Supplement 1 [file NIHPP2025.10.15.25338051v2-supplement-1.pdf]

medRxiv preprint doi: https://doi.org/10.1101/2025.10.15.25338051; this version posted November 1, 2025. The copyright holder for this preprint (which was not certified by peer review) is the author/funder, who has granted medRxiv a license to display the preprint in perpetuity.
It is made available under a CC-BY 4.0 International license .

Supplementary Information

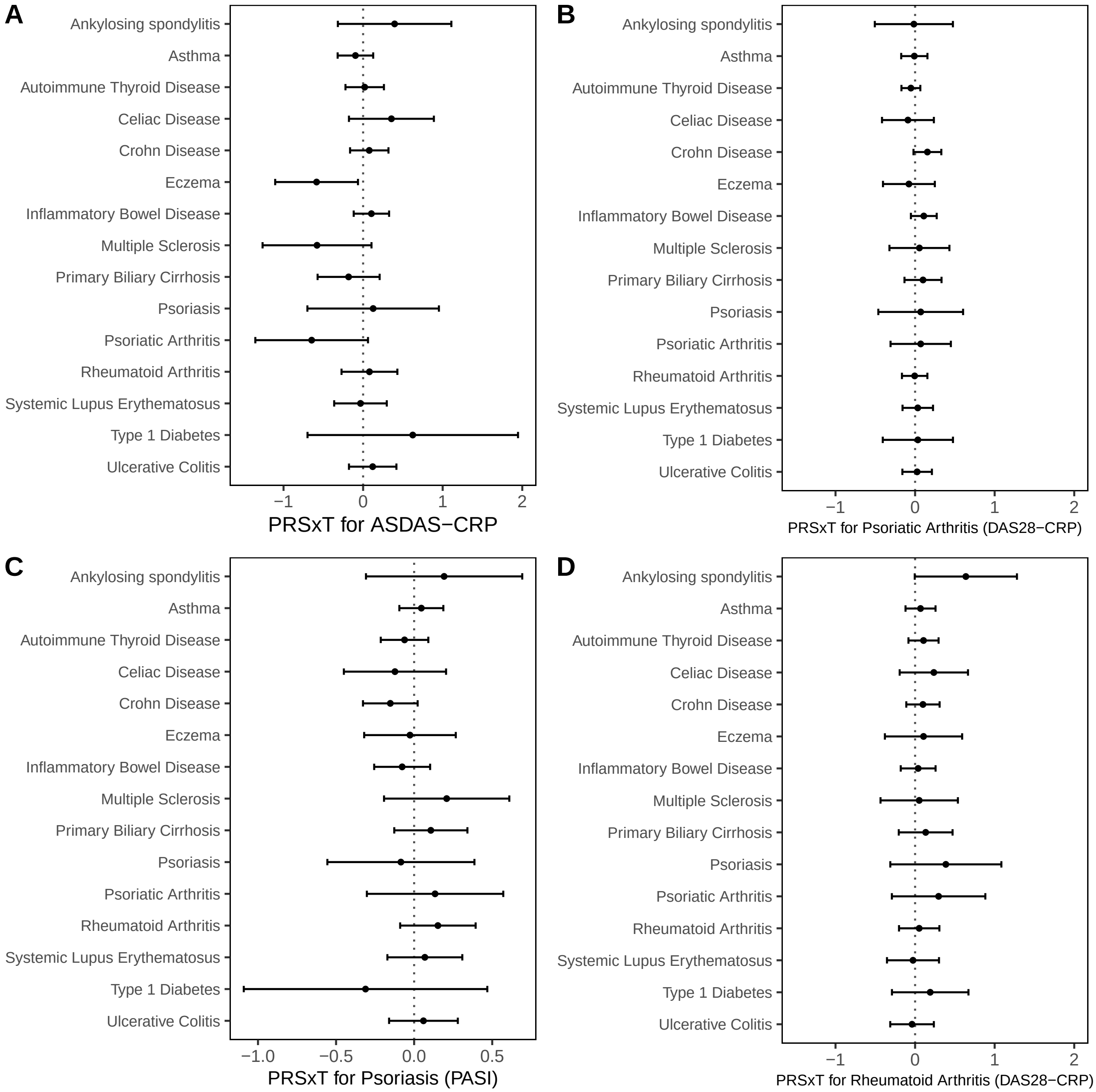

Figure S1. Polygenic risk score by treatment interaction effects (PRS×E) primary outcome for four diseases using PRS for immune-mediated diseases. Points represent estimated interaction effect sizes and horizontal lines denote 95% confidence intervals. The vertical dotted line marks the null (no interaction).

medRxiv preprint doi: https://doi.org/10.1101/2025.10.15.25338051; this version posted November 1, 2025. The copyright holder for this preprint (which was not certified by peer review) is the author/funder, who has granted medRxiv a license to display the preprint in perpetuity.
It is made available under a CC-BY 4.0 International license .

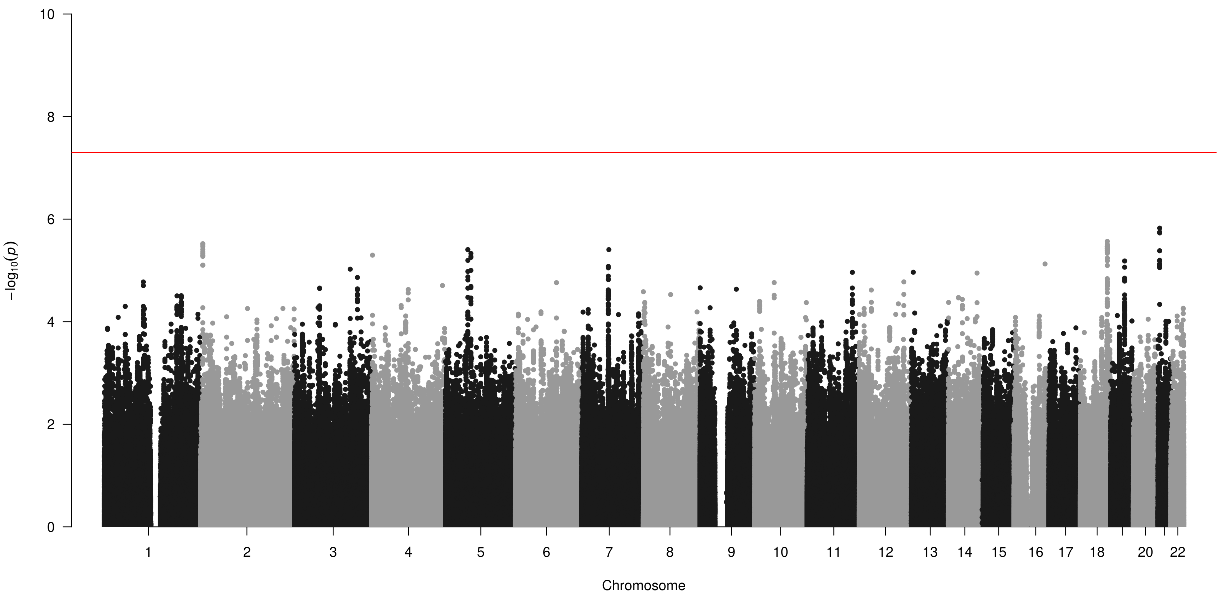

Figure S2. Manhattan plot for genome-wide genotype-by-treatment interaction results in Lung Health Study. The treatment is bronchodilator. The outcome is the changes of FEV1 (Volume that has been exhaled at the end of the first second of forced expiration) from baseline. The red line means P = 5e-8.
